# Updated View on Kidney Transplant from HCV-Infected Donors and DAAs

**DOI:** 10.3390/pharmaceutics13040496

**Published:** 2021-04-06

**Authors:** Fabrizio Fabrizi, Roberta Cerutti, Carlo M. Alfieri, Piergiorgio Messa

**Affiliations:** 1 Division of Nephrology, Dialysis and Kidney Transplant, Ca’ Granda IRCCS Foundation and Maggiore Policlinico Hospital, 20137 Milano, Italy; roberta.cerutti@policlinico.mi.it (R.C.); carlo.alfieri@policlinico.mi.it (C.M.A.); piergiorgio.messa@policlinico.mi.it (P.M.); 2 Department of Clinical Sciences and Community Health, University of Milan, 20137 Milan, Italy

**Keywords:** antiviral agents, HCV viremia, hepatitis C, kidney donors, kidney transplant

## Abstract

Background: The discrepancy between the number of potential available kidneys and the number of patients listed for kidney transplant continues to widen all over the world. The transplant of kidneys from hepatitis C virus (HCV)-infected donors into HCV naïve recipients has grown recently because of persistent kidney shortage and the availability of direct-acting antiviral agents. This strategy has the potential to reduce both waiting times for transplant and the risk of mortality in dialysis. Aim: We made an extensive review of the scientific literature in order to review the efficacy and safety of kidney transplant from HCV-viremic donors into HCV naïve recipients who received early antiviral therapy with direct-acting antiviral agents (DAAs). Results: Evidence has been rapidly accumulated on this topic and some reports have been published (*n* = 11 studies, *n* = 201 patients) over the last three years. Various combinations of DAAs were administered—elbasvir/grazoprevir (*n* = 38), glecaprevir/pibrentasvir (*n* = 110), and sofosbuvir-based regimens (*n* = 53). DAAs were initiated in a range between a few hours before renal transplant (RT) to a median of 76 days after RT. The sustained virological response (SVR) rate was between 97.5% and 100%. A few severe adverse events (SAEs) were noted including fibrosing cholestatic hepatitis (*n* = 3), raised serum aminotransferase levels (*n* = 11), and acute rejection (*n* = 7). It remains unclear whether the AEs were related to the transmission of HCV, the use of DAAs, or kidney transplant per se. It appears that the frequency of AEs was greater in those studies where DAAs were not given in the very early post-kidney transplant phase. Conclusions: The evidence gathered to date encourages the expansion of the kidney donor pool with the adoption of HCV-infected donor organs. We suggest that kidney transplants from HCV-viremic kidneys into HCV-uninfected recipients should be made in the context of research protocols. Many of the studies reported above were externally funded and we need research generating “real-world” evidence. The recent availability of pangenotypic combinations of DAAs, which can be given even in patients with eGFR < 30/min/1.73 m^2^, will promote the notion that HCV-viremic donors are a significant resource for kidney transplant.

## 1. Introduction

Hepatitis C virus (HCV) infection is recognized a public health concern affecting around 3% of people worldwide. HCV mainly targets the liver and chronic HCV frequently gives cirrhosis, liver failure, and hepatocellular carcinoma. HCV can also give extrahepatic manifestations (around 40% of the infected population) such as kidney injury, insulin resistance, and accelerated atherosclerosis [1].

The frequency of HCV infection remains high in patients with chronic kidney disease (CKD) and it has been calculated that around 10% of kidney transplant candidates are infected with chronic HCV [1]. Waiting times for kidney transplants from deceased donors commonly exceed 5 years in many countries of developed world; although the death risk is reducing among patients on the waitlist for a kidney transplant, 5% to 10% of eligible patients die each year while on the waiting list for a kidney transplant [2]. The number of patients on the waiting list for kidney transplant in the USA was 91,657 as of 31 December 2020 based on the Organ Procurement Transplantation Network (OPTN) data; the number of patients who received a kidney transplant during 2019 in the USA was 23,401 [3]. This disparity explains the long waiting times for a kidney transplant and numerous strategies that have been explored to expand access to kidney transplants in the developed world. It has been suggested that the increase of the deceased donor pool through the utilization of older donors, donation after circulatory death, and the public health service has increased the number of risk donors. 

Kidneys from HCV-viremic donors have been underused in the past. Many were discarded, and a minority was directed for HCV-positive recipients. The recent availability of direct-acting antiviral agents (DAAs) supported the widespread treatment of patients on the transplant waiting list leading to a decrease in HCV-viremic patients listed and transplanted. The option of kidneys from HCV-positive donors in HCV naïve recipients has been explored over the last few years because of various reasons, including the recent approval of direct-acting antiviral agents against HCV. DAAs have made HCV a curable infection, with frequencies of a sustained viral response in more than 95% of patients, even among transplant recipients of solid organs. Various approaches should be considered for preventing the consequences of HCV in recipients of kidneys from HCV-viremic donors: pre-emptive (antiviral therapy with DAAs initiated early post-renal transplant (RT), once viremia in the recipient is confirmed), prophylactic (DAAs initiated before transplant), or delayed (DAAs given a few weeks or months after kidney transplant). 

The aim of this narrative review is to address the pros and the cons of kidney transplants from HCV-infected donors; we also provide details on the most recent advances that have been obtained in this field.

## 2. Natural History of HCV and RT

It is well known that HCV infection is associated with poor patient/graft survival after a kidney transplant. We recently published a systematic review with a meta-analysis of observational clinical studies (*n* = 18 studies, *n* = 133,350 unique patients who underwent kidney transplant), the aggregate estimate for the adjusted relative risk (aRR) of the death rate due to any cause was 1.85 with a 95% confidence interval (CI) of 1.49; 2.31 (*p* < 0.0001) among anti-HCV-positive patients in comparison to anti-HCV-negative kidney transplant patients [4]. 

We stratified all-cause mortality with the adoption of the random-effects model; the liver disease-related mortality and the cardiovascular mortality were greater in HCV-positive patients than HCV-negative patients after RT, and the unadjusted odd ratio (OR) was 11.6 (95% CI, 5.5; 24.4) (*p* < 0.0001) and 2.15 (95% CI, 1.58; 2.91) (*p* < 0.0001), respectively [4]. 

In addition, an adverse impact of HCV upon graft survival after RT has been observed in previous studies; the overall estimate for the adjusted relative risk (RR) of all-cause graft loss was 1.76 (95% CI, 1.46; 2.11) (*p* < 0.0001) in anti-HCV-positive recipients compared with anti-HCV-negative kidney recipients [4]. HCV infection has been reported as a risk factor for the development of proteinuria after RT. Chronic infection with HCV was considered an important agent of tubulointerstitial nephritis in a large case-control survey [5]; however, HCV-related glomerulonephritis is the most frequent kidney disease among HCV-infected patients who have undergone kidney transplantation. 

Several glomerular lesions have been observed in HCV-positive patients after RT; de novo or recurrent membranoproliferative glomerulonephritis, commonly but not invariably in the context of cryoglobulinemic disease, and membranous nephropathy are the most common lesions [6,7].

Various factors have been advocated in order to explain the adverse impact of HCV on survival after RT-progressive liver disease, de novo post-transplant diabetes mellitus, and chronic allograft nephropathy, among others, appear more commonly in anti-HCV-positive recipients compared with matched-HCV-negative recipients [7]. Some authors have observed greater blood levels of calcineurin inhibitors due to impaired liver metabolism among HCV-infected kidney transplant recipients and consequent nephrotoxicity post-transplant [7]. Chronic hepatitis C is currently considered a non-conventional (but modifiable) factor for progression and incidence of chronic kidney disease in the adult general population [8].

## 3. Kidney Transplantation from HCV-Positive Donors (Early Evidence)

As mentioned above, the survival of patients with persistent HCV replication after kidney transplant is lower in comparison with HCV-negative kidney transplant recipients. However, patients with end-stage renal disease and HCV infection show better survival after RT than if they had remained on regular dialysis [9]. Thus, the best choice for end-stage renal disease (ESRD) patients with HCV infection remains kidney transplantation. 

Kidneys from HCV-infected donors have been historically underutilized. Kucirka and colleagues [10] in their investigation found that of the 93,825 deceased donors with HCV during the period 1995–2009, HCV-positive donors were 2.60 times more likely to be discarded (*p* < 0.001). Of the 6830 HCV-positive recipients, only 29% received HCV-positive kidneys. It was calculated that around 800 kidneys from donors with HCV infection have been discarded in the USA during the 2016 calendar year. Kidneys from donors with HCV infection were discarded because of the concern that no transplant center would accept these; alternatively, these organs were given to kidney transplant recipients already infected with HCV. According to the Eurotransplant database, between 2017 and 2019, 128 HCV-positive kidneys were offered and 86 of them were transplanted (discard rate, 32%). Of note, the average number of kidney transplants per year was 29 from HCV-positive and 3240 from HCV-negative donors, respectively [11]. 

Some authors reported on kidney transplants from HCV-positive donors into HCV-positive recipients. These investigators did not observe a negative impact on patient and graft survival in the short-term, but the time on the waiting list for a kidney transplant was reduced. On the other hand, many studies had a retrospective nature, the follow-up was short (up to 60 months), and they regarded small or single center series. In addition, the authors have not published any updated follow-up (reviewed in [12]). 

The largest experience of HCV-positive donors transplanted into HCV-positive recipients was made by Morales et al. [13,14]. In their study [14], 162 HCV-positive recipients received a kidney from HCV-positive donors (group 1) and 306 HCV-positive recipients received a kidney from HCV-negative donors (group 2), the mean follow-up was 74.5 months. There was no difference in 10-year patient survival rate, 72.7% versus 76.5% between group 1 and group 2, respectively; the 10-year death-censored graft survival was 47.0% in group 1 versus 58.5% in group 2 (*p* < 0.006). Because of these inferior allograft outcomes and the possibility of genotype superinfection, many transplant physicians have not adopted this approach. 

Cohen and coworkers [15] used national kidney transplant registry data and created a propensity score-matched group of HCV-positive recipients who received HCV-positive donor kidneys in comparison with those who received HCV-negative donor kidneys. These authors observed that kidney transplantation with an HCV-positive donor has a heightened death risk, with a hazard ratio (HR) of 1.43 (95% confidence interval (CI) 1.18–1.76; *p* < 0.001) and graft loss, with a HR of 1.39 (95% CI, 1.16–1.67; *p* < 0.001) in comparison to their counterparts, which were matched by propensity score analysis. Their conclusion was that the employment of kidneys from HCV-positive donors can reduce the time on the waiting list for kidney transplant and optimize the use of organs for all the candidates listed for a kidney transplant. Nonetheless, potential recipients need to be counseled about the higher risks associated with kidneys from HCV-seropositive donors. 

Gupta and colleagues [16] analyzed the OPTN registry (1994/2014). They compared the outcomes of HCV donor-positive/recipient-negative patients (*n* = 421) to propensity–matched HCV donor-negative/recipient-negative kidney transplants (*n* = 2105). They observed that the 5-year graft (44% vs. 66%, *p* < 0.001) and patient survival (57% vs. 79%, *p* < 0.001) were lower in the first group than in the second group. The authors emphasized the fact that detailed data on pre-transplant risk factors were missing. 

## 4. Kidney Transplantation from HCV-Positive Donors (Virology)

**Figure 1:**
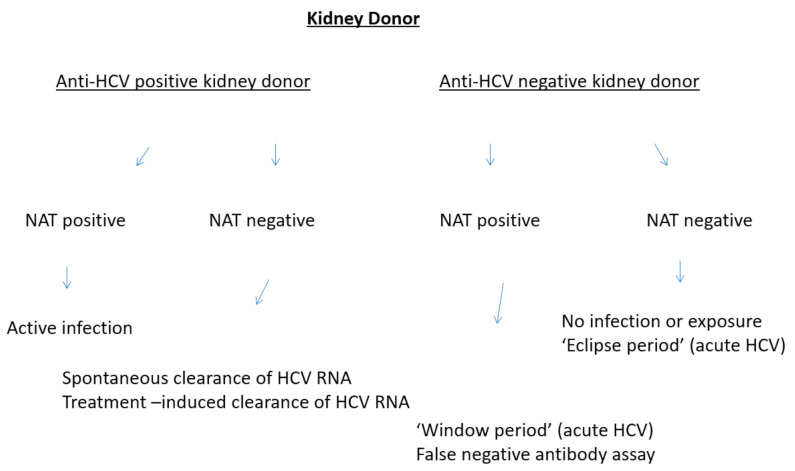
HCV diagnostic assays and kidney transplant.

The studies reported above have been hampered by a lack of differentiation between viremic (HCV RNA-positive) or non-viremic (HCV RNA-negative) anti-HCV-positive kidney donors. The United States Public Health Service guidelines currently recommend HCV nucleic acid testing (NAT) for screening of all deceased donors prior to organ procurement [17]. Testing for HCV RNA is commonly referred as HCV nucleic acid testing (NAT). Active infection occurs in anti-HCV-positive patients with detectable HCV RNA in serum; thus, NAT assesses whether active HCV infection occurs or not. NAT can be made by a branched DNA signal amplification assay (bDNA) or transcription-mediated amplification (TMA) or polymerase chain reaction (PCR). Serologic assays are utilized to detect an anti-HCV antibody in serum and make diagnosis of past or current HCV infection. The time from infection until HCV RNA is detectable in plasma by commercially available assays is referred as the pre-viremic or “eclipse” period (Figure 1). The time from the initial HCV infection until detectable antibodies appear in serum is commonly referred as the “serologic window period” [18]. Compared with serology testing, NAT assays consistently reduce the “window period” between infection and detection of HCV from around 70 days to 3–5 days. It is now clear that the risk of transmission of HCV with kidney transplantation is related to the presence/absence of HCV viremia (HCV RNA).

A recent consensus conference from the American Society of Transplantation on the employment of donors with detectable serum HCV RNA in the setting of solid organ transplant suggested to replace the term “HCV-positive donor” with the term “HCV-viremic donor” [19]. Donors without HCV RNA in serum have no risk of transmission as they have no residual virus; also, kidney transplants from donors with undetectable serum HCV RNA in naïve recipients need to be considered a safe procedure. Thus, historical outcomes data of HCV-“positive” donors must be viewed as limited because they do not specifically differentiate the presence or absence of viremia [19]. 

## 5. DAAs, HCV, and Kidney Transplant

The analysis of the genome and proteins of HCV prompted the production of direct-acting antiviral agents (DAAs), which are molecules targeting some non-structural proteins of the virus with the aim to stop viral replication and infection of HCV. Four groups of DAAs exist, these are characterized by their mode of action and therapeutic goal. The classes of DAAs are as follows, non-structural proteins 3/4A (NS3/4A) protease inhibitors, NS5B nucleoside polymerase inhibitors, NS5B non-nucleoside polymerase inhibitors, and NS5A inhibitors. 

**Figure 2:**
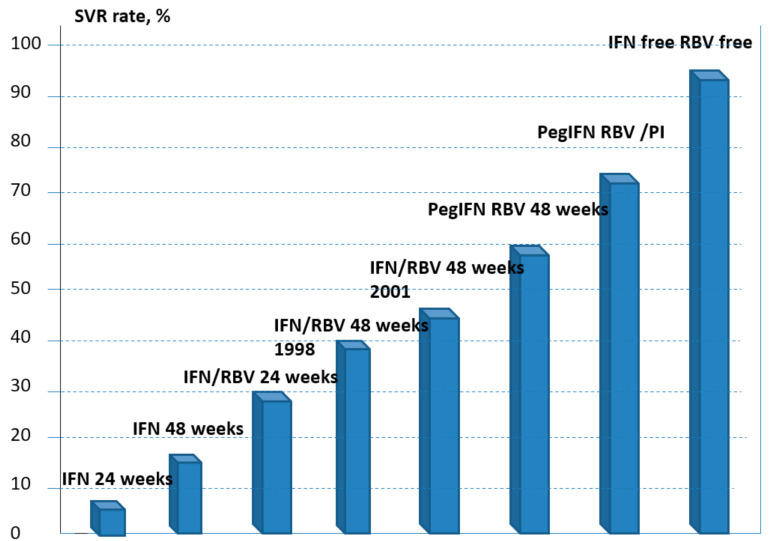
Antiviral therapy of HCV—a changing scenario.

Prior to the availability of direct-acting antiviral agents (DAAs) for treating HCV, the standard of care was Pegylated interferon (peg-IFN) in combination with ribavirin for 24 or 48 weeks. Figure 2 shows rates of SVR and anti-HCV therapies over the past two decades (native kidneys). IFN-based regimens gave limited cure rates (50–60%) and were poorly tolerated after kidney transplant because of the increased risk of acute graft rejection due to its immunomodulatory properties. Antiviral therapy based on IFN in kidney transplant recipients has been advised in specific cases, such as post-transplant cholestatic liver disease with severe course. DAAs have been recently commercialized and this consistently changed the therapy of HCV, even after solid organ transplantation.

On the grounds of the recent American Association for the Study of Liver Diseases/Infectious Disease Society of America (AASLD/IDSA) guidelines [20], some combinations of DAAs have been recommended for patients with advanced chronic kidney disease (CKD stage 4/5) and transplant recipients with HCV infection (with or without compensated cirrhosis) (Table 1). The majority of combinations for treatment of HCV are all-oral, interferon-free, and ribavirin-free, as well as being a fixed-dose combination of glecaprevir/pibrentasvir, fixed-dose combination of ledipasvir/sofosbuvir, and/or fixed-dose combination of sofosbuvir/velpatasvir. An additional choice for selected HCV genotypes is the fixed-dose combination of elbasvir/grazoprevir. The fixed-dose of sofosbuvir/velpatasvir/voxilaprevir (with or without ribavirin) has been suggested for DAA-experienced kidney transplant recipients.

As mentioned above, the therapeutic armamentarium for HCV-infected patients among kidney transplant recipients now includes sofosbuvir (SOF)-based therapies [21,22,23]. Previous studies suggested that the use of SOF-based therapies in patients with baseline estimated glomerular filtration rate (eGFR) <45 mL/min/1.73 m^2^ was associated with a greater risk of worsening kidney function in comparison with those having eGFR > 45 mL/min/1.73 m^2^ [24]. 

On the other hand, recent authors have reported that SOF use had a high efficacy and tolerability among patients with profound kidney dysfunction. In November 2019, the US Food and Drug Administration provided authorization on the use of SOF-based regimens for individuals with CKD stage 4/5 (eGFR < 30 mL/min/1.73 m^2^) irrespective if they underwent regular dialysis or not [20] (Table 1). 

Because the active metabolite of SOF is cleared by kidneys and accumulates with kidney insufficiency, patients with advanced chronic kidney disease (eGFR < 30 mL/min/1.73 m^2^) were not enrolled in initial clinical trials and were excluded from the initial labelling of the Food and Drug Administration (FDA). After its initial licensing, use of sofosbuvir in patients with advanced CKD was reported in many “real-world” studies [23] and in two phase 2 clinical trials, SOF-based combinations of DAAs in patients on maintenance dialysis have been performed [25,26]. 

## 6. Kidney Transplant from HCV-Positive/NAT-Negative (HCV RNA-Negative) Donors in Naïve Recipients

As shown in Figure 1, the presence of kidney donors who are HCV positive by antibody serologic test and negative by NAT can be related to the clearance of HCV RNA, whether spontaneous or induced by antiviral therapy. Alternatively, the occurrence of anti-HCV-positive/HCV RNA-negative individuals could be explained by false-positive antibody results or false-negative NAT results. The risk is small but exists (Table 2) [27,28,29,30,31,32,33]. However, United Network for Organ Sharing (UNOS) data show that, since 2015, no cases of HCV transmission have occurred from HCV-positive/NAT-negative kidney donors who did not have public health service (PHS) increased-risk behavior [34].

In the case of transmission of HCV by kidney transplant from anti-HCV-positive/NAT-negative donors, antiviral treatment with DAAs should be immediately conducted. We need prospective studies in order to understand how to carry out the post-transplant surveillance of patients who received kidney transplant from anti-HCV-positive/NAT-negative donors. An analysis of data from the United Network for Organ Sharing found that over a 2-year period (2015–2016), 1.8% of all donors in the USA were anti-HCV-positive/NAT-negative donors, while 4.2% were anti-HCV-positive/NAT-positive donors [35,36].

Of note, the presence of an HCV antibody with negative NAT carries a risk of transmission of HCV, which is proportional to the increased risk behavior of the donor (as an example, intravenous drug abuse), and the temporal association between serologic testing of the donor and donor behavior also plays a role. 

Kidney transplantation from HCV-positive donors (viremic and non-viremic donors) into uninfected recipients has largely increased over the last years. According to the Scientific Registry for Transplant Recipients (SRTR) data (2015–2018), HCV-positive/NAT-negative kidney donors increased from <1/month in 2015 to 26/month in 2018 in the USA. HCV-negative recipients of viremic and non-viremic kidneys spent a median (interquartile range (IQR)) of 0.7 (0.2–1.6) and 1.6 (0.4–3.5) years on the waitlist versus 1.8 (0.5–4.0) among uninfected kidney recipients of HCV-negative donors [37]. The investigators emphasized the fact that the number of HCV-positive/NAT-negative kidney donors has been driven by only a few “aggressive” transplant units. 

The epidemic of opioid users, which has been plaguing the United States over the last decade, has resulted in a consistent increase in organs for kidney transplant. Since 2000, the frequency of deaths from drug overdose has increased 137%; aside from being HCV-infected, these donors are young and commonly otherwise “healthy”. This occurs despite the increased kidney donors’ profile index in the setting of HCV infection. The kidneys from these donors have the potential to expand the donor pool given the scarcity of donated kidneys and the frequency of death on the waiting list. The possibility of transmission of other viruses (i.e., HIV) from kidney donors should also be considered in this setting. 

## 7. Kidney Transplant from HCV-Positive/NAT-Positive (HCV RNA-Positive) Donors in Naïve Recipients

Kidney transplant from HCV-viremic donors into HCV naïve recipients is an option that has been formally explored in a few clinical trials. HCV present in residual blood and fluid in the donor kidney at the time of transplantation infects the recipient kidney promptly. Early antiviral therapy with DAAs potentially gives inhibition of the HCV lifecycle, and could prevent the replication and spread of HCV after infection. Regimens provided with safety and efficacy towards all HCV genotypes, even in patients with advanced CKD, should be adopted. Overall, a total of 201 kidney transplants were collected (Table 3); as listed in Table 3 and Table 4, the majority of the reports had high efficacy and safety [38,39,40,41,42,43,44,45,46,47]. However, some studies were externally funded, and this is a source of bias [48].

The Kidney Disease: Improving Global Outcomes (KDIGO) work group recommended that “transplantation of kidneys from HCV NAT-positive donors be directed to recipients with positive NAT” [7]. The American Society of Transplantation Consensus Conference on "The Use of Hepatitis C Viremic Donors in Solid Organ Transplantation” suggested that “The prevention of HCV transmission using perioperative/postoperative DAA prophylaxis in non-viremic kidney transplant recipients of HCV-viremic donors should have institutional review board (IRB)-approved research protocols” [19]. Thus, large multi-organ studies are needed before this option becomes standard therapy. 

The DAPPeR study (also known as the Ultrashort Direct-acting Anti-viral Prophylaxis to Prevent virus transmission from hepatitis C-viremic donors to hepatitis C-negative kidney transplant Recipients study) was an open-label nonrandomized pilot single-center trial offering kidneys from HCV-viremic donors to HCV-negative kidney transplant recipients receiving an ultrashort 4-dose regimen (sofosbuvir/velpatasvir pre- and post-transplant). Additionally, 12-week DAA therapy was conducted in those patients who developed post-transplant HCV transmission [49]. The overall viral transmission rate was 12% (6/50). The conclusion of the authors was that the ultrashort course (4 days) of prophylactic DAAs significantly reduced the transmission of HCV from HCV-positive kidney donors to HCV-negative recipients, but did not eliminate it. Therefore, as they did not achieve 100% SVR, the regimen cannot be recommended as the standard of care and additional studies are needed, including a large-scale, multicenter noninferiority trial. 

The PRO-ACT study (also known as the Prevention of De Novo HCV with Antiviral HCV Therapy Post-Liver and Post-Kidney Transplant Recipients study) was recently reported. It was a multicenter survey with a prospective design which assessed the safety and efficacy of the combination of DAAs (sofosbuvir/velpatasvir for 12 weeks). Overall, 24 recipients without HCV infection underwent solid organ transplant (11 kidneys and 13 livers) from donors with an active (viraemic) HCV infection. A pre-emptive strategy was performed and antiviral therapy was initiated early in the post-transplant period, immediately after the confirmation of HCV RNA in the recipients. Antiviral therapy with SOF/velpatasvir (VEL) was started in recipients with detectable HCV RNA in serum, only when stable clinical conditions were diagnosed and a eGFR > 30 mL/min/1.73 m^2^ was observed. Nine recipients showed HCV RNA in serum on day three after the kidney transplant; one kidney transplant recipient had detectable HCV RNA in serum on day seven after the transplant. Another kidney transplant recipient did not necessitate antiviral therapy as he was persistently negative after transplantation. The median time from transplant to start of antiviral therapy was 7 (6; 12) days for liver transplant recipients and 16.5 (9.8; 24.5) days for kidney transplant recipients. Overall, 23 patients became viraemic post-transplant. All 23 treated recipients achieved SVR12. Most individuals (*n* = 21) who completed the 24 weeks of observation period achieved SVR24. No serious adverse events occurred [47]. 

## 8. Kidney Transplant from HCV/NAT-Positive Donors to Naïve Recipients: Pros and Cons

The recent availability of DAAs has led a paradigm shift in the management of post-transplant hepatitis C. DAAs give the opportunity to expand the donor pool for kidney transplant; however, the optimal timing for the initiation of DAA therapy post-kidney transplant is still controversial [50,51]. It is clear that the frequency of AEs is related to various factors including patient selection, among others; nonetheless, it appears from Table 4 that the frequency of AEs is greater when DAAs are not given in the early post-kidney transplant phase. The intense immunosuppression in the early post-transplant period has a permissive effect on viral replication with potential risks such as acute hepatitis, fibrosing cholestatic hepatitis, and immune-mediated graft dysfunction. Additional immunological risks include immune-mediated glomerulopathy, acute/chronic rejection, and antibody-mediated rejection. Therapy with DAAs administered very early after kidney transplant would likely mitigate some of these risks. The high rate of some AEs (as an example, infections by BKV and CMV) remains unclear.

The widespread treatment of patients on the transplant waiting list has led to a decrease in HCV-viremic patients listed and transplanted. Thus, the option of transplanting kidneys from donors with HCV infection in non-HCV-viremic recipients has been investigated. The opioid epidemic in the USA has resulted in a large number of drug overdose deaths and an increased number of kidneys from young and healthy donors, except for their HCV. Nonetheless, pre-emptive and prophylactic approaches are two strategies that are not easily reproducible in the “real-world” where access to DAAs can be biased by barriers such as insurance approval. Many studies reported in Table 3 and Table 4 received funding [38,39,40,42,43,47] and did not generate “real-world” evidence. It is difficult to understand whether some AEs were related to the transmission of HCV infection, use of DAAs, or kidney transplant per se. It has been recently reported the case of an HCV-viremic liver transplant recipient whose insurer did not provide initially coverage for therapy with DAAs. He developed a biopsy-proven HCV-associated glomerulonephritis and started haemodialysis. Then, he initiated therapy with DAAs with undetectable HCV viremia after 3 weeks. Dialysis was discontinued 6 weeks after the liver transplant given its decrease in serum creatinine [52]. On the other hand, prophylactic therapy with DAAs may give unnecessary treatment to those recipients without viral transmission at the time of transplant. 

The recent introduction in the market of novel combinations of DAAs with pangenotypic activity and licensed even for patients with eGFR < 30 mL/min/1.73 m^2^ [20] shouldhelp on this issue. Kidney function, indeed, is frequently impaired in the very early period post-transplant, and thus, donor genotype data cannot be unavailable in many of these clinical circumstances. These are important concerns for transplant physicians in their daily clinical activities. 

According to UNOS data, 937 transplants from HCV RNA-positive donors to HCV-seronegative recipients were performed in the USA (1 January 2015 through 31 December 2018) at 67 transplant units [3]. There was a consistent increase of these transplants over time (682 in the calendar year 2018 versus 23 in 2015). The most common organs transplanted were kidneys (*n* = 431) and liver (*n* = 244). The investigators noted that a significant variation in clinical practices at the regional and center level occurred with 469 transplants performed at a few transplant units (*n* = 8). Potluri and coworkers [53] used the OPTN registry and observed no difference in the outcome (12-month eGFR post-transplant) of HCV-viremic kidneys versus HCV-non-viremic kidneys into HCV-seronegative recipients, 66.3 ± 18.7 versus 67.1 ± 22.5 mL/min/1.73 m^2^ (NS), respectively. There was no difference in the 12-month eGFR for recipients of HCV-viremic kidneys when transplanted into HCV-seronegative versus HCV-seropositive recipients, 66.5 ± 18.8 versus 71.1 ± 18.9 mL/min/1.73 m^2^ (NS), respectively. These findings clearly promote the idea that the use of HCV-viremic donors has the potential to expand the kidney pool in a safe and effective way. 

## 9. Conclusions

Kidney transplant is still the treatment of choice for patients with end-stage kidney disease and active HCV infection. Kidney transplant from HCV-viremic donors is currently promoted by various factors including the shortage of kidney donors, and the availability of DAAs. Kidney transplant from HCV-viremic donors into HCV naïve recipients has the potential to expand the donor pool and to reduce the times in the waiting list. Some studies which highlight such strategy have been published recently. Transplanting HCV-viremic kidneys into HCV-uninfected recipients should be made in the context of research protocols; antiviral therapy should be given early to reduce the risk of HCV infection post-transplant. The advent of pangenotypic combinations of DAAs that can be administered to patients with an eGFR below 30 mL/min/1.73 m^2^ can facilitate the adoption of kidneys from HCV-viremic donors into HCV naïve transplant recipients. 

## Figures and Tables

**Table 1:** Combinations of direct-acting antiviral agents (DAAs) for therapy of hepatitis C virus (HCV) in patients with chronic kidney disease (CKD) stage 3/4 (estimated glomerular filtration rate (eGFR) < 30 mL/min/1.73 mq^2^) (dialysis dependent or not).

DAAs	Target Population (with or without Compensated Cirrhosis)
Glecaprevir (300 mg)/ pibrentasvir (120 mg) (12 weeks)	Treatment-naïve and (non-DAA) treatment-experienced patients HCV genotype 1, 2, 3, 4, 5, and 6
Ledipasvir (90 mg)/ sofosbuvir (400 mg) (12 weeks)	Treatment-naïve and (non-DAA) treatment-experienced patients HCV genotype 1, 4, 5, and 6
Sofosbuvir (400 mg)/ velpatasvir (100 mg) (12 weeks)	Treatment-naïve and (non-DAA) treatment-experienced patients HCV genotype 1, 2, 3, 4, 5, and 6
Elbasvir (50 mg)/ grazoprevir (100 mg) (12 weeks)	Treatment-naïve and (non-DAA) treatment-experienced patients HCV genotype 1 or 4

**Table 2:** Kidney transplant from anti-HCV-positive/non-viremic (HCV nucleic acid testing (NAT)-negative) donors into HCV-negative recipients: transmission of HCV.

Authors	HCV Transmission	Reference Year	Country
Tokumoto, T., et al. [27]	0/1	2000	Japan
Cruzado, J., et al. [28]	0/1	2013	Spain
Nowak, K., et al. [29]	0/21	2017	Germany
De Vera, M., et al. [30]	0/32	2018	USA
Dao, A., et al. [31]	1/40	2019	USA
Franco, A., et al. [32]	0/7	2019	Spain
Crismale, J., et al. [33]	0/4	2019	USA

**Table 3:** Kidney transplant from donors with HCV-viremia (HCV NAT-positive) into HCV-negative kidney recipients and DAAs: clinical studies.

Authors	Outcome, SVR	DAAs	DAAs Initiation
Durand, C., et al. [EXPANDER] (2018)	100% (10/10)	Elbasvir/grazoprevir ± Sofosbuvir	A few hours before RT
Reese, P., et al. [THINKER] (2018)	100% (20/20)	Elbasvir/grazoprevir	Day 3 post-RT
Friebus-Kardash, J., et al. (2019)	100% (7/7)	Sofosbuvir/ledipasvir (*n* = 4) Sofosbuvir/velpatasvir (*n* = 3)	7 days (median) post-RT
Franco, A., et al. (2019)	100% (4/4)	Glecaprevir/pibrentasvir	6 h before RT
Crismale, J., et al. (2019)	100% (7/7)	Sofosbuvir-based (*n* = 5) Glecaprevir/pibrentasvir (*n* = 2)	40 days (median) post-RT
Sise, M., et al. (2020)	100% (8/8)	Elbasvir/grazoprevir	A few hours before RT
Sise, M., et al. [MYTHIC] (2020)	100% (30/30)	Glecaprevir/pibrentasvir	2–5 days post-RT
Molnar, M., et al. (2020)	100% (53/53)	Glecaprevir/pibrentasvir (*n* = 47) Sofosbuvir/velpatasvir (*n* = 5) Sofosbuvir/ledipasvir (*n* = 1)	76 days post-RT
Feld, J., et al. (2020)	100% (10/10)	Glecaprevir/pibrentasvir + ezetimibe	6–12 h before RT
Kapila, N., et al. (2020)	97.6% (41/42)	Sofosbuvir/ledipasvir (*n* = 24) Glecaprevir/pibrentasvir (*n* = 17) Sofosbuvir/velpatasvir (*n* = 1)	72 days (median) post-RT
Terrault, N., et al. [PROACT] (2020)	100% (10/10)	Sofosbuvir/velpatasvir	16.5 days (median) post-RT

**Table 4:** Transplantation from kidney donors with HCV-viremia into HCV-negative kidney recipients and DAAs: clinical studies.

Authors	Donor HCV Genotypes	Adverse Events
Durand, C., et al. [EXPANDER] (2018)	1a (*n* = 3), 1a/3 (*n* = 1) 2 (*n* = 1), 3 (*n* = 1) Not Determined (*n* = 4)	Raised aminotransferase levels (*n* = 1) DGF (*n* = 4)
Reese, P., et al. [THINKER] (2018)	1a (*n* = 17)	Raised aminotransferase (*n* = 5) Proteinuria (*n* = 1) De novo DSA (*n* = 4)
Friebus-Kardash, J., et al. (2019)	1a (*n* = 2), 1b (*n* = 2) 3a (*n* = 1)	Arterial hypertension (*n* = 3) Sleep disorder (*n* = 1)
Franco A, et al. (2019)	1b (*n* = 3), 1a (*n* = 2)	Raised aminotransferase
Crismale, J., et al. (2019)	1a (*n* = 4), 1b (*n* = 2) 3 (*n* = 5)	Pruritus (*n* = 1)
Sise, M., et al. (2020)	1a (*n* = 6)	Raised aminotransferase (*n* = 3) BKV (*n* = 1) GI abnormalities (*n* = 1) Renal vein thrombosis (*n* = 4) Lymphocele (*n* = 4)
Sise, M., et al. [MYTHIC] (2020)	1a (*n* = 13), 2 (*n* = 1) 4 (*n* = 1)	BKV (*n* = 5) CMV (*n* = 1) Infections (*n* = 4) GI abnormalities (*n* = 5) CV events (*n* = 4) Urinary tract abnormalities (*n* = 3) DGF (*n* = 2) Acute cellular rejection (*n* = 3)
Molnar, M., et al. (2020)	1a (*n* = 34), 1b (*n* = 1) 2 (*n* = 3), 3 (*n* = 15)	De novo DSA (*n* = 16) BK viremia (*n* = 18) CMV viremia (*n* = 32) FCH (*n* = 1) Acute rejection (*n* = 4) DGF (*n* = 3)
Feld, J., et al. (2020)	NA	Raised liver enzymes (*n* = 1) Diarrhea (*n* = 1) Prostatitis (*n* = 1) Graft hydronephrosis (*n* = 1)
Kapila, N., et al. (2020)	1 (*n* = 3), 1a (*n* = 38), 2 (*n* = 6) 3 (*n* = 8), 4 (*n* = 3), 1a/3 (*n* = 1) 1b (*n* = 1), 2/3 (*n* = 1) *	FCH (*n* = 2)
Terrault, N., et al. [PRO-ACT] (2020)	NA	Raised aminotransferase levels (*n* = 4)

Adverse events related to study participation (either from HCV-viremia or antiviral therapy). FCH = fibrosing cholestatic hepatitis, CMV = cytomegalovirus, DGF = delayed graft function. * Data regarding 61 patients.

## Abbreviations

AASLD	American Association for the Study of Liver Diseases
AE	Adverse Events
AH	Arterial Hypertension
AZA	Azathioprine
bDNA	Branched DNA signal amplification
CI	Confidence intervals
CKD	Chronic kidney disease
CKD-EPI	Chronic kidney disease epidemiology collaboration equation
CMV	Cytomegalovirus
CNI	Calcineurin inhibitor
DAA	Direct-acting antiviral agent
DCV	Daclatasvir
DDI	Drug-drug interaction
DM	Diabetes mellitus
DSV	Dasabuvir
eGFR	Estimated glomerular filtration rate
EOT	End-of-treatment
ESRD	End-stage renal disease
FDA	Food and Drug administration
GI	Gastrointestinal
GN	Glomerulonephritis
HBV	Hepatitis B virus
HCV	Hepatitis C virus
HD	Haemodialysis
IDSA	Infectious Disease Society of America
IFN	Interferon
IQR	Interquartile range
IRB	Institutional review board
ITT	Intention-to-treat
KDIGO	Kidney Disease: Improving Global Outcomes
LDV	Ledipasvir
LT	Liver transplant
MMF	Mycophenolate mofetil
mTOR	Mammalian target of rapamycin
NA	Not available
NAT	Nucleic acid testing
OBV	Ombitasvir
OPTN	Organ Procurement Transplantation Network
OR	Odds ratio
PCR	Polymerase chain reaction
PegIFN	Pegylated interferon
PHS	Public health service
PI	Protease inhibitors
PTV	Paritaprevir
R	Ritonavir
RBV	Ribavirin
RR	Relative risk
RT	Renal transplant
RRT	Renal replacement therapy
SAE	Serious adverse event
SOF	Sofosbuvir
SVR	Sustained Virological Response
TAC	Tacrolimus
TMA	Transcription-mediated amplification
UNOS	United Network for Organ Sharing
USA	United States of America
VEL	Velpatasvir
WKS	Weeks
W12	12 weeks after antiviral therapy ended
